# Pedagogical impact of integration of musculoskeletal anatomy blended learning on physiotherapy education

**DOI:** 10.3389/fmed.2023.1260416

**Published:** 2023-10-12

**Authors:** Arnaud Delafontaine, Gabriel Saiydoun, Jérôme Frigout, Laurent Fabeck, Olivier Degrenne, François-Régis Sarhan

**Affiliations:** ^1^Université Libre de Bruxelles, Brussels, Belgium; ^2^CIAMS, Univ. Paris-Sud., Université Paris-Saclay, Orsay, France; ^3^CIAMS, Université d’Orléans, Orléans, France; ^4^ASSAS, Ecole de Rééducation, Département international et Recherche, Villa Thoréton, Paris, France; ^5^Unisurg, Paris, France; ^6^Department of Cardiac Surgery, Henri Mondor University Hospital, Créteil, France; ^7^Créteil, UFR Médecine-Pharmacie, University of Paris-Est Créteil, Créteil, France; ^8^Biomedicale, IMRB, Inserm, Institut Mondor de Recherche Biomédicale, Faculté de Santé de Créteil, Institut Mondor de Recherche Biomédicale, Creteil, France; ^9^Department of Cardiovascular and Thoracic Surgery, Pitié-Salpêtrière University Hospital, Assistance Publique-Hôpitaux de Paris-Sorbonne University, Paris, France; ^10^I3SP Laboratory, Department of Sports Science and Physical Education, Université de Paris Descartes, Paris, France; ^11^LIRTES, Université Paris-Est Créteil, Créteil, France; ^12^Physiotherapy School, Centre Hospitalier Universitaire Amiens – Picardie, Amiens, France; ^13^UR CHIMERE, Université de Picardie Jules Verne, Amiens, France

**Keywords:** physiotherapy student, blended learning, traditional teaching, musculoskeletal anatomy, physiotherapy education

## Abstract

**Background:**

In physiotherapy education, blended learning is recognized to be more effective compared to traditional teaching. The aim of this study was to assess the consequences of a musculoskeletal anatomy blended learning program on skills developed by students.

**Methods:**

We conducted an observational retrospective monocentric study in a French physiotherapy school named “X.” Ninety-two first-year students in the 2017–18 baseline group (students with traditional face-to-face learning), and ninety-eight first-year students and ninety-five second-year students in the 2018–19 and 2019–20 blended learning experimental groups was included. A success rate of the anatomy final written exam, defined by the percentage of students scoring 50% or above, was analyzed between 2017 and 2020. We also evaluated the pedagogical value of musculoskeletal e-learning and its usefulness for preparing the student for their anatomy final written exam at «X».

**Results:**

We observed an improvement in the success rate of the anatomy final written exam between the 2017–18 baseline group, 2018–19 and 2019–2020 experimental groups during first (Kruskal–Wallis = 74.06, df = 2, *p* < 0.001) and second semester (Kruskal–Wallis = 173.6, df = 2, *p* < 0.001). We obtained a data survey and questionnaire response rate of 74% (*n* = 89/120) for the 2018–19 and 62% (*n* = 72/116) for the 2019–20 experimental groups. Concerning questionnaire response, they were no significant statistical difference between 2018–19 and 2019–20 experimental groups.

**Conclusion:**

Blended learning could improve student success rate of the anatomy final written exam and learning of professional physiotherapy skills.

## 1. Introduction

### 1.1. Background

**Figure 1:**
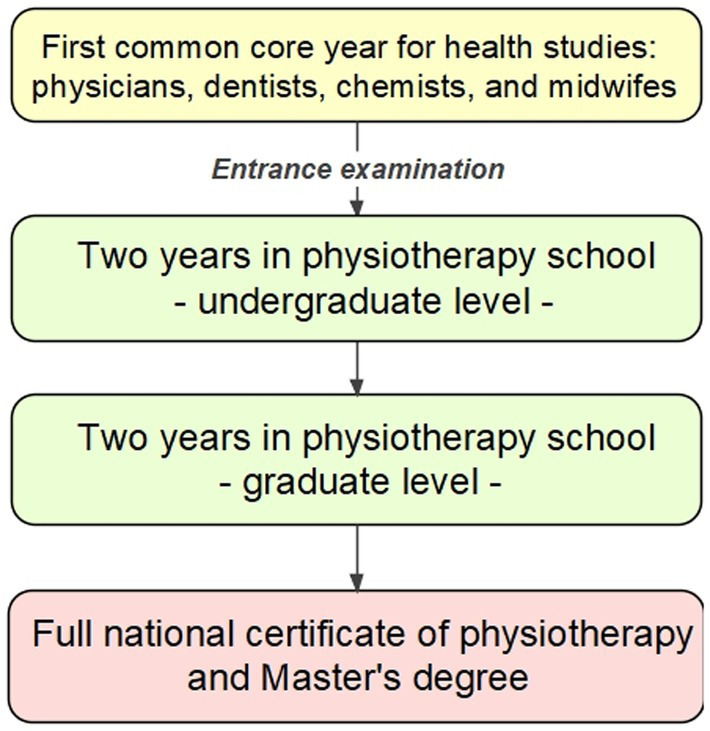
Physiotherapy program over five years: a first, common year is followed by 4 years of specialization in physiotherapy.

Physiotherapy education in France has transformed since 2015, shifting toward a university degree program (1, 2). Historically, physiotherapy schools were separate from universities and awarded a “Diplôme d’Etat de Masseur-Kinésithérapeute (DEMK)” after a three- or four-year program. The new system is a five-year university-based program (1, 3, 4) (Figure 1), starting with a common year of university alongside other healthcare programs, followed by specialized physiotherapy education.

During the first year, students study general courses, including chemistry, physics, biochemistry, and others (5, 6). Second-year admission is based on an entrance exam for physiotherapy, medicine, dentistry, pharmacy, or midwifery. Years two to five are taught at physiotherapy schools, which collaborate with universities to establish conditions for earning European Credits (ECTS) and a Master’s degree.

The healthcare reform facilitated e-learning adoption, a popular and efficient teaching mode in the medical field, especially amid the coronavirus disease of 2019 (COVID-19) pandemic (7, 8).

In fact, Rossettini et al. (9, 10) underlined that, in post-COVID-19 period, physiotherapy educators had to implement digital education into entry-level physiotherapy education, especially to deal with social inequality and evaluation of students.

For instance, “X” physiotherapy school in the Paris region had to develop innovative ways to teach musculoskeletal anatomy with limited hours alongside medicine students at Paris-Saclay University’s Faculty of Medicine (11).

E-learning, particularly blended learning, has become the standard in medical and physiotherapy education [(12–16); Rossettini et al. (9, 10); (17, 18)]. Blended learning enhances learning outcomes, satisfaction, and attitudes among physiotherapy students (19–21) but may not significantly improve clinical practice (22, 23).

E-learning improves inter-professional collaboration among medical, nursing, physiotherapy, and occupational therapy students (24) and enhances anatomy learning when combined with traditional teaching methods (25–27). It benefits students who require visual and kinesthetic learning, like physiotherapists (28). However, despite of these benefits, a recent systematic review (29) shows that most studies used non-validated tools in order to quantify the improvement of digital health competencies due to digitalization among healthcare professionals.

### 1.2. Objectives

In “X” physiotherapy school, e-learning was introduced to complement traditional classroom-based anatomy courses. This study aims to assess the impact of musculoskeletal anatomy blended learning on final exam performance compared to traditional teaching methods. The hypothesis is that blended learning will lead to improved success rates (30).

Overall, the healthcare reform in France has paved the way for the integration of e-learning in physiotherapy education, enhancing learning experiences and academic outcomes (31, 32).

## 2. Materials and methods

### 2.1. Study design

We used data from a monocentric observational retrospective study on physiotherapy students. The STROBE guidelines were adhered to by the methodology of the article (33).

### 2.2. Setting

The study was conducted in a French public physiotherapy school (i.e., named “X”) in partnership with Paris-Saclay University. About 450 students are enrolled in “X” four-year physiotherapy study program. Every year, approximately 100 first-year students are admitted to “X” for the first common year of health education.

### 2.3. Participants

We considered data from second- and third-year physiotherapy students. This study was conducted over three university years from September 2017 to July 2020. Repeat students and students with flexible work arrangements (i.e., top-level athletes) were excluded. The study complied with the standards set by the Declaration of Helsinki (34). All participants gave written informed consent after being instructed as to the nature and purpose of the study, which was approved by the local ethics committee of Paris-Saclay University under registration number CER-Paris-Saclay-2020-095.

A baseline group (2017–18 baseline group) was formed of second-year physiotherapy students who attended only in-person gross anatomy courses with no specific physiotherapy musculoskeletal blended learning in anatomy (from September 2017 to July 2018).

Two experimental groups (2018–19 and 2019–20 experimental groups) were formed. Both groups had previously attended in-person gross anatomy courses and specific physiotherapy musculoskeletal anatomy blended learning (from September 2018 to July 2019, and from September 2019 to July 2020).

### 2.4. Intervention

Thirty-two musculoskeletal anatomy blended learning units were created for the second year of physiotherapy studies at “X.” The learning objectives were to focus on musculoskeletal anatomy to complement the gross anatomy studies delivered at Paris-Saclay University. These studies were dedicated to myology, osteology and arthrology of the upper and lower limbs. To standardize and homogenize the content of these blended learning units, all were prepared and recorded by the same individual (Professor of musculoskeletal anatomy, with 10 years of teaching experience). None of the students received direct in-person instruction from this individual. The 32 blended learning units were peer reviewed by the authors of this article for consistency and quality of content. All blended learning units were broadcast by the intranet server of “X” physiotherapy school (i.e., digital teaching platform accessible at http://www.learneos.fr) and freely accessible for each student on their own school’s account.

To control bias, all of the blended learning units had the same structure and duration (i.e., 30 min) and were pre-recorded with the same teacher’s voice. The blended learning units were composed of anatomy bullet text, 2D/3D musculoskeletal anatomy pictures (i.e., illustrations, diagrams and 3D models) and cadaveric musculoskeletal images. No e-video was included in the blended learning units.

### 2.5. Variables

#### 2.5.1. Primary outcome

The anatomy skills developed by the students were assessed through first- and second-semester final examination results. We compared the results of students with traditional face-to-face learning (2017–18 baseline group) with those of students having completed the blended learning program (2018–19 and 2019–20 experimental groups). Success in the anatomy teaching unit was defined by the rules of the physiotherapy program: students were required to obtain a score of 10 out of 20 (or 50%) on the first multiple-choice exam. Multiple-choice exam is composed of 30 to 40 multiple-choice questions (i.e., 4 possible answers for each question) based on musculoskeletal anatomy program of the first-year physiotherapy students (i.e., myology, osteology and arthrology of the upper and lower limbs).

#### 2.5.2. Secondary outcomes

An investigation field was made through an online data survey to evaluate the pedagogical value of musculoskeletal blended learning and its usefulness for preparing the student for their anatomy final written exam at “X” physiotherapy school.

The retrospective target and eligible population and the eligibility criteria corresponded to second- to third-year physiotherapy students at “X” physiotherapy school over three university years from September 2017 to July 2020.

Concerning the sources and methods of selection, all the participants were recruited through the survey. Participants were able to complete the survey at any time during the period mentioned above. All data were self-reported by the participants. The survey was anonymous, and data confidentiality was assured in accordance with the European General Data Protection Regulation.

The 2018–19 and 2019–20 experimental group students were asked to evaluate the pedagogical value and interest of blended learning as an effective tool for preparing written semester exams (Table 1).

**Table 1:** 2018–19 and 2019–20 experimental group student’s questionnaire assessment.

Assessment items	Questionnaire assessment
Pedagogical value of musculoskeletal anatomy e-learning	Question 1. Did you find blended learning support useful in learning musculoskeletal anatomy? Only one answer possible: □ Yes □ No
	Question 2. Could blended learning replace in-person classroom lectures? Only one answer possible: □ Yes □ No
	Question 3. For the next cohort of second-year physiotherapy students, should in-person anatomy classes be maintained in addition to blended learning? Only one answer possible: □ Yes □ No
	Question 4. Would you like to receive anatomy musculoskeletal e-video learning resources? Only one answer possible: □ Yes □ No
	Question 5. For you, is it “essential” to include cadaveric musculoskeletal images in blended learning? Only one answer possible: □ Yes □ No
	Question 6. For you, is it “essential” to practice “cadaveric dissection” during physiotherapy studies at “X“? Only one answer possible: □ Yes □ No
Usefulness of musculoskeletal anatomy e-learning for preparing for the final written exam	Question 7. Was blended learning useful for preparing for the final written exam? Only one answer possible: □ Yes □ No
	Question 8. Was blended learning useful in preparing for your future profession as a physiotherapist? Only one answer possible: □ Yes □ No
	Question 9. Do you believe that blended learning allowed you to learn musculoskeletal anatomy more easily than in-person learning at Paris-Saclay University’s Faculty of Medicine? Only one answer possible: □ Yes □ No
	Question 10†. For the next cohort of second-year physiotherapy students, do you believe it essential to maintain anatomy classes with in-person teaching in addition to blended learning? Only one answer possible: □ Yes □ No

† Assessment specific to 2019–20 (3rd year) experimental group.

### 2.6. Bias

Considering our study design, several potential biases must be underlined. First, we cannot exclude a social desirability bias (where respondents to surveys tend to answer in a manner they feel will be seen as favorable by others) and a selection bias given the way we recruited participants through monocentric training institute.

### 2.7. Statistical methods

A descriptive analysis was performed to determine the average score of each blended learning unit. The Shapiro–Wilk W test was used to evaluate each variable for normality and established that nonparametric statistic tests should be used (Shapiro test with *p* < 0.001). The mean score during both semesters concerned was compared for 2017–18 and 2019–20 with Kruskal-Wallis test. The mean score between groups (i.e., 2017–18 baseline group, 2018–19 and 2019–20 experimental groups) was compared with Mann–Whitney test. Statistical analysis was performed using JASP® (Version 0.14.1, Amsterdam, Netherlands). A Fisher’s exact test was performed to determine the association between dependent and independent variables. Statistical analysis was performed using SPSS statistical software (Version 23.0 for Mac, SPSS, Inc., Chicago, IL). The level of significance was established at *p* < 0.05.

## 3. Results

### 3.1. Participants and descriptive data

**Figure 2:**
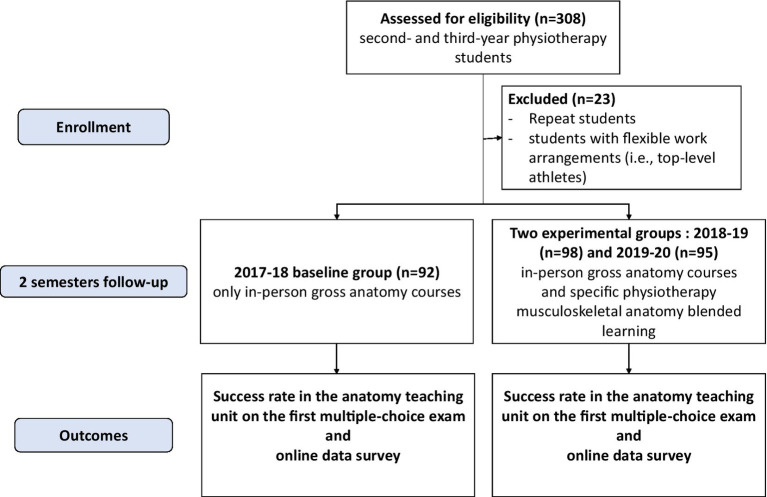
Flowchart of the study.

A total of 308 students were included in the three cohorts, with 23 students being excluded due to academic repetition or having flexible work arrangements (Figure 2).

We considered ninety-two first-year students (19 ± 1 years old; 52 males and 39 females) in the 2017–18 baseline group, and ninety-eight first-year students (20 ± 1 years old; 49 males and 49 females) and ninety-five second-year students (21 ± 2 years old; 52 males and 43 females) in the 2018–19 and 2019–20 experimental groups.

### 3.2. Main results

#### 3.2.1. Primary outcome

For the main outcome, we observed an improvement in the success rate of the anatomy final written exam between the 2017–18 baseline group and the 2018–19 and 2019–2020 experimental groups. Success rate is defined by the percentage of students scoring 50% or above. Table 2 shows an improved success rate of the anatomy final written exam for both semesters studied.

**Table 2:** Success rate of the anatomy final written exam for both semesters studied of the 2017–18 baseline group, and of the 2018–19 and 2019–20 experimental groups.

	First semester				Second semester			
Year	n	n Success 1st exam	n Success 2nd exam	Success rate^¥^ (%)	n	n Success 1st exam	n Success 2nd exam	Success rate^¥^ (%)
2017–18	92	45	37	89	98	55	13	69
2018–19	98	84	12	98	99	38	43	82
2019–20	95	84	11	100	96	96	n/a	100

n, number of students.

¥ Success rate is defined by the percentage of students scoring 50% or above.

**Figure 3:**
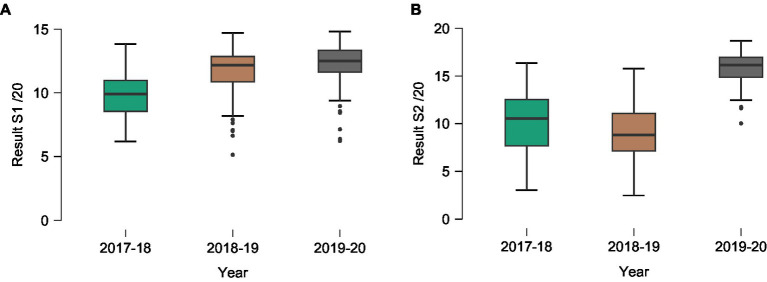
Mean result and standard deviation of students for first **(A)** and second **(B)** semesters in anatomy without (2017–18 baseline group) and with blended learning (2018–19 and 2019–20 experimental groups). S1: first semester; S2: second semester.

For the first semester, the mean score results to final exam of the 2017–18 baseline group, and of the 2018–19 and 2019–20 experimental groups improved (Kruskal-Wallis = 74.06, df = 2, value of *p*<0.001) (Table 3 and Figure 3).

**Table 3:** Results of final exam of the 2017–18 baseline group, and of the 2018–19 and 2019–20 experimental groups. n: number of students.

		First semester			Second semester			
Year	n	Mean result*	Median (IQR)	*p*-value Kruskal–Wallis test	n	Mean result*	Median (IQR)	*p*-value Kruskal–Wallis test
2017–18	92	9.87	9.91 (2.47)	Kruskal-Wallis = 74.06, df = 2, value of *p*<0.001	102	10.19	10.54 (5.03)	Kruskal–Wallis = 173.6, df = 2, value of p<0.001
2018–19	98	11.70	12.19 (2.02)		99	8.96	8.82 (4.06)	
2019–20	95	12.29	12.58 (1.77)		96	15.77	16.14 (2.09)	

IQR, interquartile range.

* Min: 0; Max: 20.

For the second semester, the mean score results to final exam of the 2017–18 baseline group, and of the 2018–19 and 2019–20 experimental groups improved (Kruskal-Wallis = 173.6, df = 2, value of *p*<0.001) (Table 3 and Figure 3).

The mean score results to final exam of 2018–19 experimental group significantly decreased (Mann–Whitney = 6195.5, value of p<0.001) compared to the 2017–18 baseline group.

The mean score results to final exam of 2019–20 experimental group significantly increased (Mann–Whitney = 163.0, value of *p*<0.001) compared to 2018–19 experimental groups.

#### 3.2.2. Secondary outcomes

For the secondary outcome, we analyzed the pedagogical value and usefulness of for musculoskeletal blended learning for preparing for the “X” final written exam. We obtained a data survey and questionnaire response rate (Figure 3; in line with 2018–19 and 2019–20 experimental group students questionnaire assessment of Table 1) of 74.2% (total of 89/120 data surveys and responses available) for the 2018–19 experimental group, and 62.1% (total of 72/116 data surveys and responses available) for the 2019–20 experimental group. Concerning questionnaire response, they were no significant statistical difference between 2018–19 and 2019–20 experimental group students.

##### 3.2.2.1. Pedagogical value

**Figure 4:**
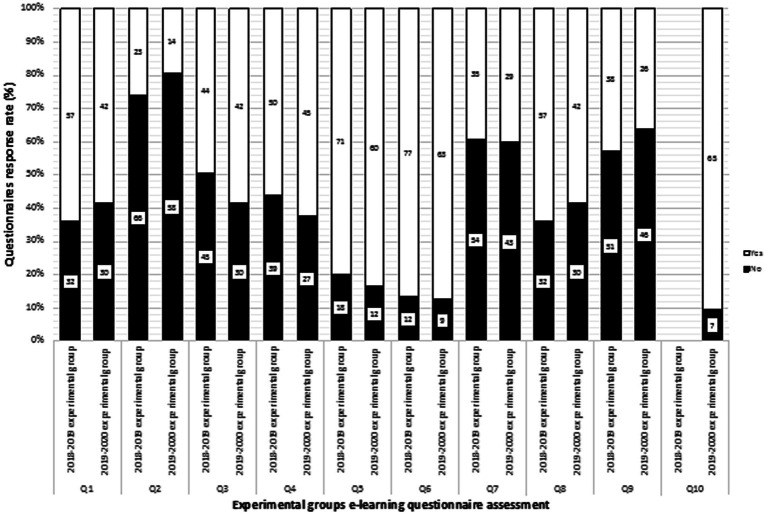
Comparison of 2018–19 (n = 89) and 2019–20 (n = 72) students questionnaire response rate. Q: question in line with Table 1. The number of each respondent (i.e., “yes” and “no”) is indicated on each column from Q1 to Q10.

We observed that 74% (*n* = 66/89) of 2018–19 experimental group students and 80% (*n* = 58/72) of 2019–20 experimental group students said that blended learning could not replace in-person classroom lectures (see Q1 of Figure 4).

However, 64% (n = 57/89) of 2018–19 experimental group students and 58% (n = 42/72) of 2019–20 experimental group students found that blended learning support is useful for learning musculoskeletal anatomy (see Q2 of Figure 4).

##### 3.2.2.2. Usefulness of blended learning for preparing for final exam

The assessment of musculoskeletal blended learning as an efficient tool for preparing for the “X” final written exam showed that 61% (n = 54/89) of 2018–19 experimental group students and 60% (*n* = 43/72) of 2019–20 experimental group students do not consider blended learning anatomy to be useful in preparing for the final written exam (see Q7 of Figure 4).

## 4. Discussion

### 4.1. Key results

The primary outcome showed a significant improvement in the success rate of the anatomy final written exam for both semesters studied in the 2018–19 and 2019–20 experimental groups compared to the 2017–18 baseline group. Mean score results for the final exam were significantly better in the experimental groups than in the baseline group. The secondary outcomes explored the pedagogical value and usefulness of musculoskeletal blended learning for preparing for the anatomy final written exam. A majority of students found blended learning helpful for learning musculoskeletal anatomy, although they believed it could not fully replace in-person classroom lectures for anatomy instruction. Overall, the study suggests that implementing blended learning can lead to improved exam performance and is considered valuable for learning musculoskeletal anatomy by many students, though not as a complete substitute for traditional classroom lectures.

In physiotherapy education, the use of blended learning increases: knowledge, practical skills acquisition (19, 21), satisfaction/attitude in physiotherapy students (20) and workload (22). In line of the literature, the primary outcome of the study suggests that the addition of e-learning to traditional learning improved the success rate of physiotherapy students in gross anatomy. This improvement may be attributed to the better organization of students facilitated by the creation of a note-taking network specific to the e-learning course. In other words, the implementation of e-learning resources and tools had a positive impact on students’ ability to organize and comprehend the subject matter, leading to improved academic performance. On the other hand, the secondary outcome reveals that despite the positive effects observed in the primary outcome, the majority of students did not consider e-learning anatomy useful in preparing for the final written exam. They expressed a belief that in-person classroom lectures were irreplaceable when it came to studying anatomy. Several factors may contribute to the differences between the primary and secondary outcomes. Firstly, Learning Preferences: Students have diverse learning preferences and styles. While some may find e-learning resources effective for organizing and understanding the material, others may prefer the traditional classroom setting and face-to-face interactions for learning complex subjects like anatomy (35, 36). The secondary outcome highlights the continued preference for in-person lectures among some students. Secondly, Perceived Value: Students’ perceptions of the value and relevance of e-learning resources may vary (37). Even though the primary outcome suggests improved success rates, students may not perceive the e-learning anatomy resources as directly contributing to their performance in the final exam. They may prioritize the interactive nature, real-time feedback, and immediate clarification opportunities provided by in-person lectures. Thirdly, Comfort and Familiarity: Students may be more comfortable and familiar with traditional classroom lectures due to their prior educational experiences. They might have developed effective study strategies and routines around in-person lectures, making it difficult for them to fully embrace and utilize the e-learning resources for exam preparation (38). Finally, this difference car be explained by Subject Complexity: Anatomy is a complex subject that often requires hands-on learning, visual aids, and direct interaction. Some students may perceive e-learning resources as inadequate in providing these elements, which can lead to their preference for in-person classroom lectures (39). It is important to note that these differences in perception and preference between the primary and secondary outcomes do not negate the positive impact of e-learning observed in the primary outcome. Instead, they highlight the need for considering individual learning styles and preferences when implementing blended learning approaches and designing effective e-learning resources for anatomy education (40, 41).

### 4.2. Limitations

The limitations of this retrospective study are the risk of memory bias for 2019–20 experimental student’s group and the absence of a musculoskeletal anatomy e-learning performance assessment such as the one conducted by Laveneziana et al. (42). However, it is the first pedagogical study assessment performed in a French physiotherapy school. Pedagogical management needs to be studied further scientifically, especially because of the recent creation of the National University Council (“CNU 91”) for Reeducation and Rehabilitation Sciences approved by the French Ministry. Moreover, this questionnaire has been translated to English and the lack of piloting, forward and backward translation is a severe limitation. The methodology used for the questionnaire could be notably enhance by using the Likert scale for example. The questionnaire was more related to a field investigation. The reliability and validity were not tested. Further studies need to take into account some confusing factor with a stratified analysis (e.g., the level of exam difficulty, the level of the students, the used of incidental/parallel learning of anatomy other than the e-learning resource, the individual duration time according to learn during the pandemic, …)”.

### 4.3. Interpretations

#### 4.3.1. About the overall education process

As previously described by Freeman et al. (43), the results of this study show that e-learning added to traditional learning of gross anatomy (i.e., blended learning) could improve the success rate of physiotherapy students. It seems that the student’s anatomy results are optimal two years after the introduction of e-learning in physiotherapy school. It is probably linked to a better organization of physiotherapy students thanks to the creation of note-taking network specific to the e-learning course, which benefited the students from the following group (see results of group 2019–20 on Figure 2).Our results confirm that anatomy e-learning cannot replace in-person classroom lectures, although the majority of students consider it a useful teaching format for anatomy (44). The new findings of this study are as follows: (1) the majority of students do not consider e-learning anatomy useful in preparing for the final written exam and would like to have anatomy video e-learning as a complement. In fact, the use of video e-learning support at the beginning of the first semester could help health students to improve their university exam performance (42). A recent systematic review, written by Noetel et al. (45), underlines that videos are unlikely to be detrimental and usually improve student learning at university. A recent meta-analysis, conducted by Fontaine et al. (46) also suggests that adaptive e-learning (notably with videos) appear effective in improving skills in health students and professionals by generating less cognitive load.

#### 4.3.2. Unexpected results

However, it should be noted that the decrease in mean score results for the final exam of the 2018–19 experimental group, compared to both the 2017–18 baseline group and the 2019–20 experimental group, raises interesting questions. Several potential factors may explain these results, particularly within the 2018–19 experimental group. One possible explanation could be a lack of organizational strength and insufficient sleep among the students, with reported sleep durations as short as 3–4 h per night (47). Such sleep deprivation has been linked to cognitive impairment and decreased academic performance. Additionally, it’s important to consider the overall performance of the student cohort during that period. It is plausible that the 2018–19 group, as a whole, experienced lower academic performance compared to other cohorts. This could be attributed to various factors, such as changes in the curriculum, teaching methods, or even external influences like personal circumstances or distractions. Nonetheless, it is crucial to acknowledge that our study did not assess the quality of students’ lives during the research period. Research has shown that factors like lifestyle, stress levels, and overall well-being can significantly impact the academic performance of health students (48). Therefore, it is possible that the observed decline in exam scores could be related to such unmeasured variables. Future studies should consider exploring the influence of these factors to gain a comprehensive understanding of the performance variations observed among different student groups. That is why while the decrease in mean scores for the 2018–19 experimental group is evident, it is essential to recognize the limitations of our study and the potential influence of various factors on academic performance. Further investigation considering factors like sleep patterns, overall well-being, and students’ quality of life would provide valuable insights into the observed outcomes.

#### 4.3.3. About student’s satisfaction

The overall quality of e-learning was good based on the ratings assigned by the majority of students. E-learning is of professional value for physiotherapy students as it can improve their ability to anticipate clinical situations and physiotherapy tasks. Mazzoleni et al. (49) showed that students (72% of 2034 users) are generally satisfied with the blended learning content and that it contributes to the improvement of results in continuing medical education. For corroborate this result, Jebraeily et al. (50) have reported, through a recent qualitative study, that the productive lecturer-student interactions were improved with the virtual component, students yet questioned the lack of sufficient and on-time feedback from the lecturers on their activities. They suggest that the use of different types of interactions should still be monitored and promoted through online discussions, on-time feedbacks, and forums to compensate for the lack of rich face-to-face interactions that take place for clarifications or confirmations in classroom teaching. The authors propose a systematic evaluation of blended medical education from lecturers and student’s viewpoint using the following items: Strengths, Weaknesses, Opportunities, and Threats (SWOT). In a pedagogical way, they propose the analysis of SWOT items and it mindful consideration in each context, in order to adopt the right implementation and management strategies to achieve sustainable benefits for students and pedagogical team.

Students consider the presence of *in-vivo* anatomical dissection pictures to be indispensable in the e-learning support and describe anatomical dissection as fundamental during physiotherapy studies. The study found that the adoption of blended learning in physiotherapy education assisted physiotherapy students to perform better in exams and develop relevant skills. It is suggested that the use of video e-learning support at the beginning of the first semester could help healthy students to improve their university exam performance.

#### 4.3.4. Educational considerations

From a pedagogical point of view, similar results were highlighted who stressed the need to combine e-learning with in-person courses (i.e., blended learning) to limit the risk of students “dropping out” [Varga-Atkins et al., 2005; (26, 27)].

In our observation, the majority of the 2018–19 and 2019–20 experimental student’s groups [i.e., 2018–19 experimental group students: 74.2% (n = 72) and 2019–20 experimental student’s groups: 80.6% (n = 76)] do not believe that e-learning anatomy will replace in-class anatomical courses in the future. This corroborates the results of Ruiz et al. (32) who point out that students do not see e-learning as a replacement for traditional classroom training, but as complementing it.

E-learning is a professional value for physiotherapy students. For example, it is demonstrated that e-learning improves the ability to anticipate clinical situations and physiotherapy tasks (51). Our findings corroborate the results of Riffell & Merrill (52) supporting the fact that e-learning must be included in the educational program right from the beginning of the university program. This encouraged the «X» anatomy teaching team to add e-learning to first-year physiotherapy studies.

Therefore, the «X» anatomy teaching team will maintain this teaching format with 2018–19 experimental student’s groups. The results of a meta-analysis (53) comparing an online versus in-person learning situation showed that students with online learning achieved better results than those receiving in-person instruction. However, students who received combined learning (i.e., online and in-person) achieved the best results. Our results corroborate this observation, with 90.3% (n = 85) of 2019–20 experimental student’s groups considering it essential to maintain anatomy classes with in-person teaching at «X» in addition to anatomy e-learning for the next cohort of 2018–19 experimental student’s groups.

Laveneziana et al. (42) showed that 50% of second-year medical students believe that e-learning video sessions could replace the traditional classroom (i.e., in-person). This result is also found in the literature (54–56). Actually, most of pedagogical teams (e.g., physiotherapy and medicine), work with e-video-based lectures, coupled to peer-mentoring (57), for enhancing the anatomy skills (58–60) of students and their diagnosis (54, 55).

These observations remain to be qualified since 74.2% (n = 72) of 2018–19 experimental student’s groups and 80.6% (*n* = 76) of 2019–20 experimental student’s groups think that e-learning cannot replace traditional classroom courses. This can be explained by the fact that, pedagogically, physiotherapy students are required to develop their anatomical knowledge better in a sensitivo-sensory practical aspect (i.e., touching, massaging, manipulating, observing anatomical structures) compared to second-year students of medicine (61).

Indeed, physiotherapy students’ use up to six palpatory skills (62) integrated in a somato-psychic educational process (63). The use of e-video would also improve test preparation (56, 64) and optimize students’ learning pattern (54, 65). Guy et al. (57) showed that students using e-video media available in their curriculum in addition to their traditional course materials achieved better results, and there was a linear relationship between the number of e-video viewed by the students and their results on the exams.

Physiotherapy students (2018–19 and 2019–20 experimental student’s groups) assign particular importance to anatomical dissection and dissection pictures as a teaching aid. This corroborates the meta-analysis conducted by Yammine and Violato (66) where the use of physical models was shown to provide statistically superior results in short- and long-term overall anatomical knowledge acquisition and spatial tracking, compared to 3D modeling alone. The use of 2D and 3D interactive anatomical support allows students, regardless of their spatial anatomical modeling ability (67), to progress in learning anatomy (68), including by combining these two supports (69). However, a literature review conducted by (70) shows that the use of 3D anatomical support versus traditional teaching is equal. Therefore, for physiotherapy students, anatomy blended learning must present cadaveric musculoskeletal images paired with dissection in the laboratory. These results are in line with the literature (71) which considered cadaveric dissection as an educational tool for anatomical sciences improving teamwork, self-reflection, interprofessional communication skills, and ethical qualities. Varying the modes of anatomical learning would optimize visuo-constructive capabilities and visual–spatial anatomical identification, as is the case in surgery (72).

### 4.4. Generalisability

The results of this study need to be reinforced by a multinational study. We can also mention the epistemological limitations inherent in the quantitative approach of this type of study (observational survey study). Indeed, purely quantitative approaches restrict the field of analysis and do not allow for an in-depth understanding of the behavior of individuals. The complementary use of qualitative approaches, allowing a broader, more complete, more global, and richer understanding of the phenomena studied, is to be sought.

The need to enhance anatomy education in physiotherapy schools was brought into focus by the global COVID-19 pandemic (73) thanks to a multidisciplinary approach (74). Further studies are required to better understand the pedagogical needs of anatomy educators and physiotherapy students in order to improve professional skills.

## 5. Conclusion

Blended learning, which combines e-learning with traditional in-person teaching, can improve the success rate of physiotherapy students, particularly in gross anatomy. The optimal results are observed two years after the introduction of e-learning, which may be due to the better organization of students thanks to the creation of note-taking networks specific to the e-learning course. However, e-learning cannot replace in-person classroom lectures, although the majority of students consider it a useful teaching format for anatomy. Most students believe that e-learning anatomy cannot replace in-class anatomical courses in the future, but it can complement them. The study also shows that the presence of *in-vivo* anatomical dissection pictures is indispensable in the e-learning support, and e-learning improves the ability to anticipate clinical situations and physiotherapy tasks. Furthermore, the students who received combined learning (i.e., online and in-person) achieved the best results. Therefore, the “X” physiotherapy school anatomy teaching team will maintain the blended learning format with in-person teaching at “X” in addition to anatomy e-learning for the next cohort.

In conclusion, since recent years, teaching in physiotherapy is undergoing a substantial change by the use of new teaching methods (75). Digital and massive online courses need a strong cooperation between political, scientific and professional actors (8). In this global educational context, blended teaching should be integrated into physiotherapy in the future and tested by combining/mixing teaching techniques for manual skills as well as theoretical knowledge (76–79). Also, specific outcomes (e.g., psychological, emotional) and the ability to use digital technology to self-learn and teach others must be consider for future studies (29).

## Data availability statement

The original contributions presented in the study are included in the article/supplementary material, further inquiries can be directed to the corresponding author.

## Ethics statement

The studies involving humans were approved by the Ethics Committee of Paris-Saclay University under registration number CER-Paris-Saclay-2020-095. The studies were conducted in accordance with the local legislation and institutional requirements. Written informed consent for participation was not required from the participants or the participants’ legal guardians/next of kin in accordance with the national legislation and institutional requirements.

## Author contributions

AD: Conceptualization, Data curation, Investigation, Methodology, Project administration, Supervision, Validation, Visualization, Writing – original draft. GS: Conceptualization, Validation, Visualization, Writing – original draft. JF: Conceptualization, Investigation, Validation, Writing – original draft. LF: Conceptualization, Validation, Writing – original draft. OD: Conceptualization, Data curation, Methodology, Validation, Writing – original draft. F-RS: Conceptualization, Data curation, Formal analysis, Methodology, Validation, Writing – original draft.
